# Clinical validation of a non-invasive electrodermal biofeedback device useful for reducing chronic perceived pain and systemic inflammation

**DOI:** 10.1007/s42000-019-00098-5

**Published:** 2019-02-25

**Authors:** George P. Chrousos, Dario Boschiero

**Affiliations:** 1 Choremeion Research Laboratory, First Department of Pediatrics, National and Kapodistrian University of Athens Medical School, Athens, Greece; 2 Unit of Translational and Clinical Research in Endocrinology, National and Kapodistrian University of Athens Medical School, Athens, Greece; 3 BioTekna - Biomedical Technologies, Marcon, Venice, Italy

**Keywords:** Electrodermal biofeedback, Chronic inflammation, Pain, Stress, RegMatEx, C-reactive protein

## Abstract

**Background:**

This study was performed to evaluate the potential clinical usefulness of a new non-invasive electrodermal biofeedback device in reducing perceived pain levels and chronic systemic inflammation.

**Materials and methods:**

This multicenter study was designed and coordinated by BioTekna, included 20 general practice medical centers, took place between June 2010 and January 2011, and was validated clinically at the National and Kapodistrian University of Athens, Greece. The study participants were 1015 Caucasian men (401) and women (614), while the placebo-treated controls were 950 Caucasian men (500) and women (450). Patients were aged between 30 and 86 years (average age about 50 years) and all suffered from chronic pain and other medically unexplained symptoms (MUS). The RegMatEx electrodermal biofeedback device (brand BioTekna - Biomedical Technologies, Marcon, Venice, Italy) was used to evaluate the clinical efficacy of electrodermal biofeedback in reducing the level of pain perceived by decreasing the chronic systemic inflammation of the subjects. All subjects received 6 × 30 min sessions of electrodermal or placebo biofeedback given twice a week over 3 weeks. Perceived pain was evaluated using the Numeric Rating Scale (NRS) for pain, while systemic inflammation was examined with measurements of morning circulating C-reactive protein (CRP) concentrations.

**Results:**

Perceived pain in the treatment group was significantly lessened in the NRS scale (*p* < 005), while circulating CRP concentrations were also decreased (*p* < 0.05). Parallel placebo studies showed no changes in perceived pain or morning serum CRP concentrations.

**Discussion:**

Use of the electrodermal biofeedback RegMatEx device was associated with reduced pain perception and decreased chronic systemic inflammation, with stability over time. This did not occur in the placebo-treated group. The symptomatology of the treated patients significantly improved in terms of pain relief as shown on the NRS scale, and this was accompanied by reported improvements in mobility, mood, and quality of life.

**Conclusions:**

The RegMatEx electrodermal biofeedback procedure is a non-invasive and easy to use therapeutic method, free of side effects, with high patient acceptability, excellent efficacy, and duration of effect, and, hence, a valuable tool in the treatment of chronic pain and inflammation.

## Introduction

This study was performed to evaluate the clinical applications of a new non-invasive electrodermal biofeedback device, which would be potentially useful in reducing perceived pain levels and chronic systemic inflammation. According to the definition of the International Association for the Study of Pain (1986) and the World Health Organization (WHO), pain “is an unpleasant sensory and emotional experience associated with tissue damage, either ongoing or potential, or described in terms of damage”. Pain represents a composite of (a) a perceptive component (nociception), i.e., the sensorial modality that allows reception and transmission of stimuli potentially harmful to the organism and to the central nervous system, and (b) an experiential part which is the psychological state connected to the perception of an unpleasant feeling. Thus, pain is a defense reaction and, therefore, physiologically a vital symptom, as it represents a warning signal to the brain of tissue injury, essential to avoid or minimize damage. The pain becomes pathological when it is maintained over time, losing its initial teleology and becoming a disease, i.e., a chronic pain syndrome [1]. Pain is thus a fundamental function for the survival of the individual, with pain-sensing receptors able to identify various types of dangerous stimuli, mechanical, chemical, and thermal.

Epidemiologic investigations [2, 3] were conducted in various European countries, including Italy. In the latter country, chronic pain afflicts 1 in 4 people (about 15 million Italians), for an average period of 7.7 years, while 1/5 patients had been suffering from pain for over 20 years. These data highlight the magnitude of the problem, which is particularly impactful in patients with chronic diseases, such as rheumatoid arthritis, osteoarthritis, fibromyalgia, osteoporosis, chronic headaches, irritable bowel syndrome, and cancer. A recent study was performed in 13 European countries via interviews and follow-up over time in patients suffering from chronic non-cancer pain. Of these patients, 47% complained of severe intensity pain with a duration longer than 2 years [4].

The chronic pain present in degenerative, neurological, and oncological diseases, especially in their advanced and terminal stages, assumes the characteristics of systemic or global pain and is linked to major physical, psychological, and social changes, as highlighted in the WHO documents [5]. It is important always to carefully evaluate pain when treating it, because each individual reacts uniquely to a specific painful stimulus, based on previous experiences and different individual threshold levels. Pain is an unpleasant subjective experience and usually has an emotional component. Pain is measured using official scales validated by international clinical trials [6, 7]. Pain assessment is performed by asking the patient to assign a score corresponding to the tested pain. The Numeric Rating Scale (NRS) for pain is based on the use of a scale consisting of 11 degrees from 0 to 10, where 0 corresponds to the total absence of pain and 10 represents the worst pain imaginable by the patient [8, 9]. The different scales employed for pain assessment cannot, among themselves, be formally compared; generally, however, NRS is accepted as a reliable procedure.

Inflammation is a defense mechanism of the body whose goal is the elimination of the cause of the injury and the subsequent repair of the damaged tissues; it is a necessary protection mechanism; however, the persistence of even a low-grade chronic inflammatory state is a common feature of a wide range of chronic disorders and/or diseases. Low-grade chronic systemic inflammation is a widespread and rapidly increasing human disease state, linked to the modern lifestyle and environmental pollution. Low-grade inflammation has several causes, some being chronic stress, overweight/obesity, sedentary life, bad nutrition, loss of circadian rhythmicity, and poor sleep. Thus, it is a systemic inflammatory process strongly correlated with type of feeding (excessive caloric intake, latent metabolic acidosis, excessive production of insulin, imbalance of fatty acids, lack of CoQ10 and L-carnitine, and excess positive potential renal acid load (PRAL) (pro-inflammatory food), scarce or absent physical activity, chronic stress, sleep deprivation, and alterations in biological rhythms. The chronic low-grade systemic inflammation manifests with a constellation of “non-specific” symptoms (medically unexplained symptoms, MUS) that include asthenia, fatigue, daytime sleepiness, nocturnal insomnia, irritability, and difficulty in concentration. Over time, its failure to correct promotes numerous disease states, including obesity, type 2 diabetes mellitus, malignant neoplasms, and neurodegenerative diseases (in particular Alzheimer’s disease, multiple sclerosis, and Parkinson’s disease). It involves the extracellular matrix and the cells of all the tissues and organs of the body. Chronic or autoimmune persistent inflammation also leads to increased acute phase reactants, in particular of C-reactive protein (CRP). This protein is produced by the liver and secreted into the bloodstream. As the plasma concentration of CRP increases within a few hours from the beginning of an inflammatory reaction, it is particularly useful to monitor its course [10]. An increase in circulating CRP levels simply reflects an ongoing inflammatory state; this increase may be non-specific with respect to an inflammation site. Usually, in healthy subjects, CRP levels are lower than 5 mg/L [11], while, on average, they are higher in women than in men and rise with aging [12].

In the presence of chronic inflammation with altered hydroelectrolytic conditions of the extracellular environment (increase in extracellular water [ECW], loss of intracellular potassium [increase in serum potassium]), the cell membrane resting potential decreases to values less than − 70 mV, with a time trend towards 0 mV. The consequences of these changes are (a) a decrease in the ability to reach the threshold potential, (b) a reduction of the frequency of action potential, and (c) a net decrease in local and systemic hydroelectrolytic regulatory capacity with reduced functional, reparative, or conservational activities. Restoration of membrane potential and, therefore, of the normal hydroelectrolytic condition is necessary to overcome the state of chronic allostasis or, more accurately, cacostasis. Whatever method is used, there are action potentials when an externally applied stimulus has sufficient amplitude and duration to cause an ionic flow through the cell membrane and to raise its potential above the threshold value.

The RegMatEx electrodermal device uses the principle of biofeedback of the measured systemic basic and stimulated potential, which allows evocation of a greater frequency than that in the basal state of chronic inflammatory allostasis or cacostasis, inducing cellular repolarization (− 70 to − 90 mV) and stimulating the biofeedback processes of the autonomic nervous system. We hypothesized that stimulating cellular repolarization lowers pain and inflammatory processes by contributing to the control of intra- and extracellular hydroelectrolyte balance.

## Materials and methods

### Subjects

This multicenter study was designed and coordinated by BioTekna: it involved 20 general medical practice centers and took place between June 2010 and January 2011. The results were evaluated and validated by the National and Kapodistrian University of Athens, Greece.

The study participants were Caucasian men and women aged between 30 and 86 years (Tables 1, 2, 3, 4, 5, 6, 7, and 8). The number of subjects involved in the treatment arm of the study was 1015, comprising 401 males and 614 females, with an average age of 48 years. In all subjects, pain was a major feature of their lives (Table 1). The placebo study participants were also Caucasian men and women aged between 30 and 86 years also suffering from chronic pain and MUS (Table 5). The number of subjects involved was 950, composed of 500 males and 450 females, with an average age of 50 years. The placebo group underwent the same procedure as the treated group. The only variation was that the device-patient connection cable was internally silenced so as to keep all the other variables exactly the same.

**Table 1:** Distribution of chronic inflammatory diseases with presence of pain. The study participants were Caucasian men and women aged between 30 and 86 years. The number of subjects involved was 1015, comprising 401 males and 614 females, with an average age of 48 years. In all the subjects, pain was a major feature of their lives

	*M*	*F*
Rheumatoid arthritis	195	231
Osteoarthritis	65	132
Cervical pain	16	79
Fibromyalgia	45	53
Low back pain	11	21
Chronic recurrent headaches	49	68
Chronic nonspecific pains	20	30
Total	401	614

The patients involved in the study stated that the pain they experienced had a negative effect on their ability to lead a normal life: 73% of them had difficulty in performing their everyday activities, such as domestic work or family and recreational happenings, 68% were unable to perform their professional work, 46% had disruptions of their family and social relationships, 60% had poor quality of sleep, and only 41% had sexual relationships. The pain interacted with the emotional state of those affected, some of whom reported that they would prefer to die.

### Device

The RegMatEx electrodermal biofeedback device (brand BioTekna - Biomedical Technologies, Marcon, Venice, Italy) was used to evaluate the clinical efficacy of electrodermal biofeedback in reducing the level of perceived pain and chronic inflammation in the treated subjects. The device was registered in Italy at the Ministry of Health—national classification of medical devices CND: Z12062404 as a “system for the biofeedback of electrodermic signal.” All the subjects had 6 × 30 min sessions of electrodermal biofeedback over a 3-week period. The NRS pain scale and circulating C-reactive protein concentrations were used for the evaluation of perceived pain and inflammation, respectively.

### Principles of device function

The non-invasive biofeedback medical device for the regulation of cell membrane electric potentials reads the action potential differences of the human body in the least distal parts (hands) and generates feedback of potential values between the device and the patient in the most distal parts (feet). The system, through low-frequency polarized electromagnetic signals, modifies ionic mobility of the key electrolytes by stimulating active exchange through action potentials, concentration gradients, and electrostatic forces, in effect favoring the repolarization of cell membrane potentials.

### Procedure sequence

1. Systemic reading of the tissue potentials through two electrodes positioned in the wrists (mV measurement).
2. Real-time analysis of electrical potentials across a frequency spectrum range generated by intra- and extracellular ion exchanges (from 0 to 500 KHz).
3. Biofeedback for correction of the potentials measured (inversion of potentials). The RegMatEx generates potential differences, modulates membrane potentials, increases threshold potentials, and induces an autonomic nervous system reaction. These changes are thought to have the potential to reduce chronic pain and inflammation by affecting regulatory mechanisms altered by endogenous and exogenous stress stimuli.

To evaluate and validate the efficacy of biofeedback in the modulation of membrane potentials in subjects with chronic pain and inflammation, the level of pain on the NRS scale and serum CRP concentrations were evaluated. CRP was used as a nonspecific inflammation marker to determine the effectiveness of anti-inflammatory therapy. In healthy people, the mean value of CRP is low, while for most cases of chronic inflammation there are measurable increases in CRP to levels higher than 5 mg/L, with average values of about 15 mg/L.

### Protocol for the clinical evaluation of electrodermal biofeedback

Recruitment of volunteer patients and controls for the NRS interview and the morning serum CRP measurement. Patients had pain and other MUS and received a diagnosis, as shown in Tables 1, 2, 3, 4, 5, 6, 7, and 8.

**Table 2:** Distribution of the degree of chronic pain (mean values) by the NRS scale: (0 = absence of pain, 10 = worst pain). The study participants were Caucasian men and women aged between 30 and 86 years. The number of subjects involved was 1015, comprising 401 males and 614 females, with an average age of 48 years. In all the subjects, pain was a major feature of their lives

	*M*	*F*
Rheumatoid arthritis	6	7
Osteoarthritis	3	4
Cervical pain	3	5
Fibromyalgia	7	8
Low back pain	6	4
Chronic recurrent headaches	8	7
Chronic nonspecific pains	5	7

**Table 3:** Mean change in CRP levels per disorder before and after 6 × 30 min biofeedback sessions over a 3 week cycle. The study participants were Caucasian men and women aged between 30 and 86 years. The number of subjects involved was 1015, comprising 401 males and 614 females, with an average age of 48 years. In all the subjects, pain was a major feature of their lives

	Average initial CRP	Average final CRP
Rheumatoid arthritis	20.8	7.53
Osteoarthritis	8.9	4.14
Cervical pain	9.56	2.76
Fibromyalgia	31.1	8.95
Low back pain	7.9	4.02
Chronic recurrent headache	6.07	4.17
Chronic nonspecific pains	8.11	3.42

**Table 4:** Mean change of the NRS scale of the degree of chronic pain before and after the 6 × 30 min biofeedback 3 week cycle. (0 = absence of pain, 10 = worse pain). The study participants were Caucasian men and women aged between 30 and 86 years. The number of subjects involved was 1015, comprising 401 males and 614 females, with an average age of 48 years. In all the subjects, pain was a major feature of their lives

	NRS average initial		NRS average final	
	*M*	*F*	*M*	*F*
Rheumatoid arthritis	6	7	1	2
Osteoarthritis	3	4	0	0
Cervical pain	3	5	0	1
Fibromyalgia	7	8	1	1
Low back pain	6	4	1	0
Chronic recurrent headaches	8	7	2	2
Chronic nonspecific pains	5	7	0	2

**Table 5:** Distribution of chronic inflammatory diseases with presence of pain. The placebo study participants were Caucasian men and women aged between 30 and 86 years. The number of subjects involved was 950, comprising 500 males and 450 females, with an average age of 50 years. In all the subjects, pain was a major feature of their lives. The placebo group had the same procedure as the treated group. The only variant was that the device-patient connection cable was internally silenced, so as to keep all the other variables exactly the same

	*M*	*F*
Rheumatoid arthritis	200	191
Osteoarthritis	75	88
Cervical pain	24	25
Fibromyalgia	45	53
Low back pain	24	21
Chronic recurrent headaches	87	48
Chronic nonspecific pains	45	24
Total	500	450

**Table 6:** Distribution of pathologies and the degree of chronic pain (mean values) in the NRS scale (placebo group): (0 = absence of pain, 10 = worse pain). The placebo study participants were Caucasian men and women aged between 30 and 86 years. The number of subjects involved was 950, comprising 500 males and 450 females, with an average age of 50 years. In all the subjects, pain was a major feature of their lives. The placebo group had the same procedure as the treated group. The only variant was that the device-patient connection cable was internally silenced, so as to keep all the other variables exactly the same

	*M*	*F*
Rheumatoid arthritis	6	6
Osteoarthritis	4	3
Cervical pain	4	5
Fibromyalgia	7	8
Low back pain	5	4
Chronic recurrent headaches	7	7
Chronic nonspecific pains	5	6

**Table 7:** Mean change in serum CRP for each disorder before and after the cycle of 6 × 30 min biofeedback sessions (placebo group). The placebo study participants were Caucasian men and women aged between 30 and 86 years. The number of subjects involved was 950, comprising 500 males and 450 females, with an average age of 50 years. In all the subjects, pain was a major feature of their lives. The placebo group had the same procedure as the treated group. The only variant was that the device-patient connection cable was internally silenced, so as to keep all the other variables exactly the same

	Average initial CRP	Average final CRP
Rheumatoid arthritis	21.2	20.88
Osteoarthritis	8.3	8.11
Cervical pains	8.45	7.68
Fibromyalgia	29.3	30.8
Low back pain	8.2	7.61
Chronic recurrent headaches	7.2	6.8
Chronic nonspecific pains	8.9	8.1

**Table 8:** Mean change of the NRS scale of the degree of chronic pain before and after the 6 × 30min biofeedback sessions (placebo group). (0 = absence of pain, 10 = worst pain). The placebo study participants were Caucasian men and women aged between 30 and 86 years. The number of subjects involved was 950, comprising 500 males and 450 females, with an average age of 50 years. In all the subjects, pain was a major feature of their lives. The placebo group had the same procedure as the treated group. The only variant was that the device-patient connection cable was internally silenced, so as to keep all the other variables exactly the same

	NRS average initial		NRS average final	
	*M*	*F*	*M*	*F*
Rheumatoid arthritis	6	6	6	6
Osteoarthritis	4	3	3	3
Cervical pain	4	5	3	4
Fibromyalgia	7	8	7	8
Low back pain	5	4	4	4
Chronic recurrent headaches	7	7	7	6
Chronic nonspecific pains	5	6	4	6

### Patient preparation

Hydration with water equal to at least 3% of body weight from the day before the start of the first biofeedback session.

### BioFeedback cycle

Six treatments with the RegMatEx device, with two biofeedback sessions per week × 30 min each, in a nonspecific mode (preset by default).

### Data collection

Medical history and physical examination, NRS interviews, and CRP measurements were obtained at the beginning and at the end of each electrodermal biofeedback cycle.

### Data analysis

The Student’s *t* test was employed to examine the differences in scores of the NRS interviews and serum CRP before and at the end of the treatment or placebo cycle.

Exclusion criteria for participation in the electrodermal biofeedback study:

As a precaution for the safety of the patients and given the paucity of literature on electrodermal biofeedback applications, we excluded pacemaker carriers, carriers of deep neural stimulators, and epileptic subjects. Even though the technique is non-invasive, biofeedback-generated induction of action potentials might interfere with electromagnetic phenomena.

## Results

### Treatment group

The distribution of the degree of chronic pain in the patients in the treatment arm (mean values) by the NRS pain scale (0 = absence of pain, 10 = worst pain) is shown in Table 2. The mean change in CRP levels per disorder before and after 6 × 30 min biofeedback sessions over a 3-week cycle is shown in Table 3. The mean change of the NRS scale of the degree of chronic pain before and after the 6 × 30 min biofeedback 3-week cycle (0 = absence of pain, 10 = worst pain) is shown in Table 4. Thus, perceived pain in the treatment group was significantly lessened in the NRS scale (*p* < 0.005), while the circulating CRP concentrations were decreased (*p* < 0.05).

### Placebo group

The distribution of chronic inflammatory disorders with presence of pain is shown in Table 6. The mean change in serum CRP for each disorder before and after the cycle of 6 × 30 min placebo biofeedback is shown in Table 7. The mean change of the NRS pain scale of the degree of chronic pain before and after the 6 × 30 min biofeedback sessions (0 = absence of pain, 10 = worst pain) is depicted in Table 8. Perceived pain scores in the placebo group remained unchanged in the NRS scale (*p* > 0.05), while the circulating CRP concentrations were also stable during the placebo treatment (*p* > 0.05).

## Discussion

Chronic stress and inflammation in adults have been associated with MUS, including pain and increased extracellular water, abdominal obesity, osteosarcopenia, a flattened salivary cortisol rhythm, and elevated circulating CRP concentrations [13, 14]. We have hypothesized that the MUS are, in fact, a combination of stress and “sickness syndrome,” manifestations elicited by sustained activation of the stress system and the immune and inflammatory reaction [15]. The subjects included in this study had as predominant manifestations hyperalgesia and frank pain, both products of the interaction between immune mediators and the peripheral and central nervous system.

The use of the electrodermal biofeedback RegMatEx device in our subjects was associated with reduced pain perception and decreased chronic systemic inflammation, with stability over time. This did not occur in the placebo-treated group. The symptomatology of the treated patients significantly improved in terms of pain relief shown in the NRS pain test, and this was accompanied by reported improvements in mobility, mood, and quality of life. Thus, the RegMatEx electrodermal biofeedback procedure is a non-invasive and easy to use therapeutic method, free of side effects and with high patient acceptability, excellent efficacy and duration of effect, and therefore a valuable tool in the treatment of chronic pain and inflammation.

There may be conditions that can lead to lower therapeutic performance in terms of effectiveness of the above method over time. On the other hand, the technique has limitations in the following situations: (a) inaccurate reading of electrical tissue potentials due to poor water turnover (less than 2% of body weight) or with very low total body water (less than 45%), (b) reduced ion exchange from chronic use of anti-inflammatory drugs, and (c) low presence of electrolytes essential for the maintenance of action potentials due to the chronic use of diuretics (low induction of action potentials due to loss of electrolytes).

In conclusion, the electrodermal biofeedback RegMatEx device produced significant results in reducing subjective pain and objective chronic inflammation. After a 3-week cycle of six biofeedback treatments, the symptomatology of the patients significantly improved.

Simultaneously, the concentrations of circulating C-reactive protein were decreased. The RegMatEx electrodermal biofeedback device is thus a valuable tool in the treatment of both physical and mental pain related to chronic stress and inflammation. Given the validity of this method, other applications can be envisioned, both in disease and health. We have now extended this study by following the patients with the Health Questionnaire EQ-5D-5 L and with the perceived stress scale. In these patients, using a different device, namely, “PPG Stress Flow,” we are also examining heart rate variability, vagal tone, and the balance between the sympathetic and parasympathetic systems over a long period of time. It is noteworthy that vagal tone exerts systemic anti-inflammatory actions and could explain our findings, at least partially [16].
